# Supplementary figures and images for: Intramammary rapamycin administration to calves induces epithelial stem cell self-renewal and latent cell proliferation and milk protein expression
Source: PLoS One. 2022 Jun 22;17(6):e0269505. doi: 10.1371/journal.pone.0269505 (PMC9216576; doi:10.1371/journal.pone.0269505)

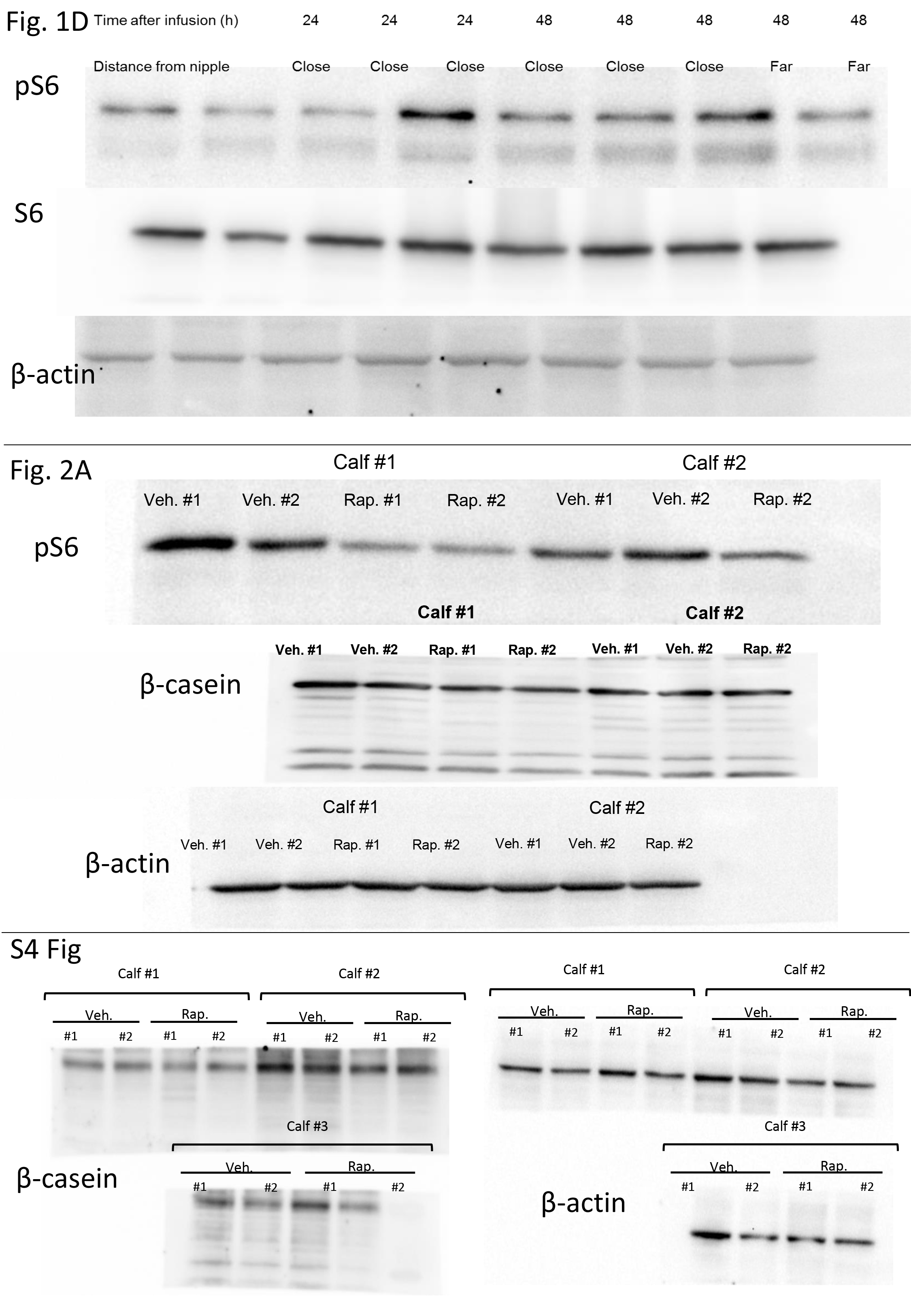

Supplement: S1 Raw images — (TIF) [file pone.0269505.s001.tif]

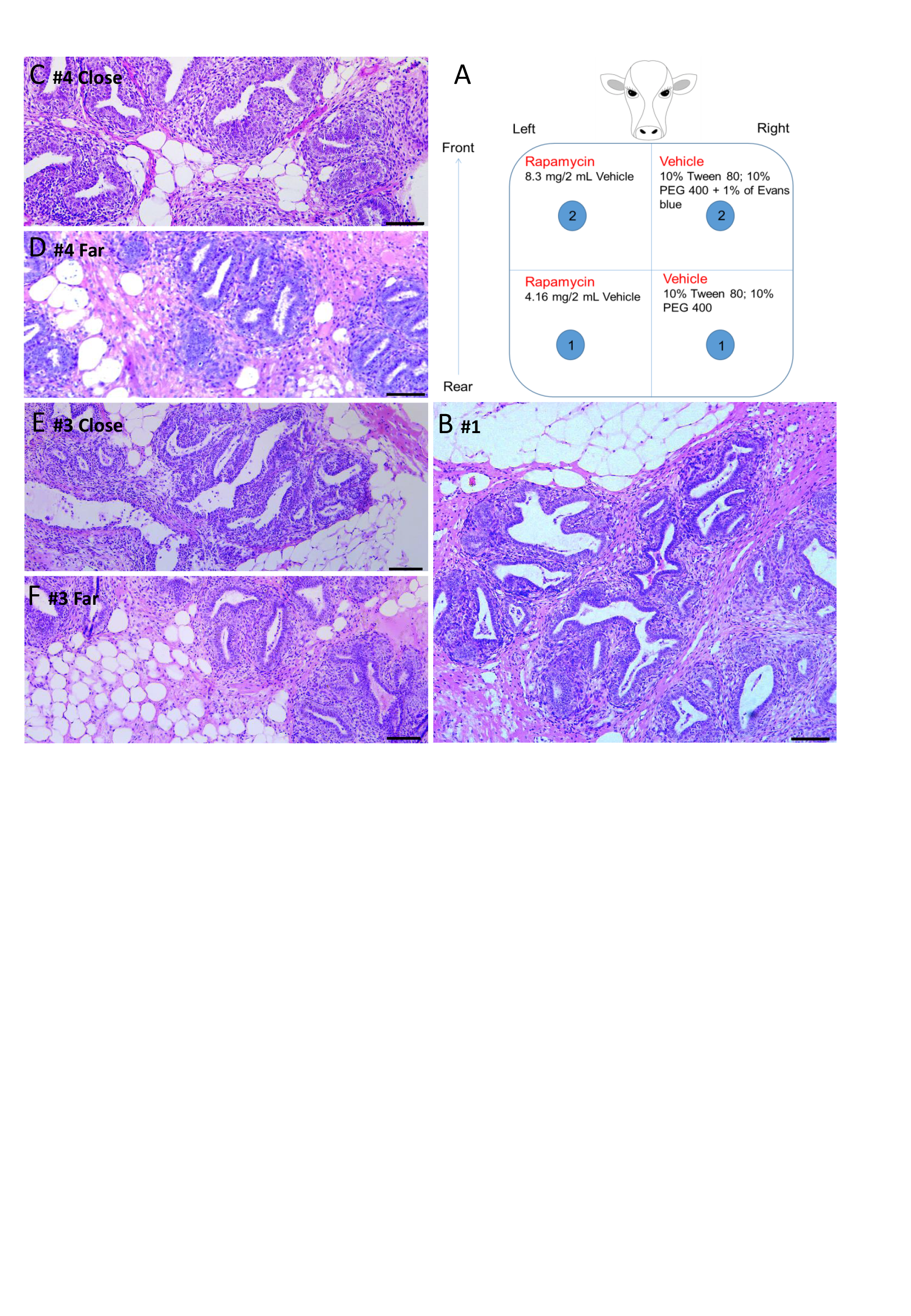

Supplement: S1 Fig — A. Designation of the bovine mammary glands for intramammary vehicle and rapamycin administration. B–F. Morphology of parenchymal regions close to and far from the nipple is not affected by rapamycin administration. Bar = 50 μm. (TIF) [file pone.0269505.s005.tif]

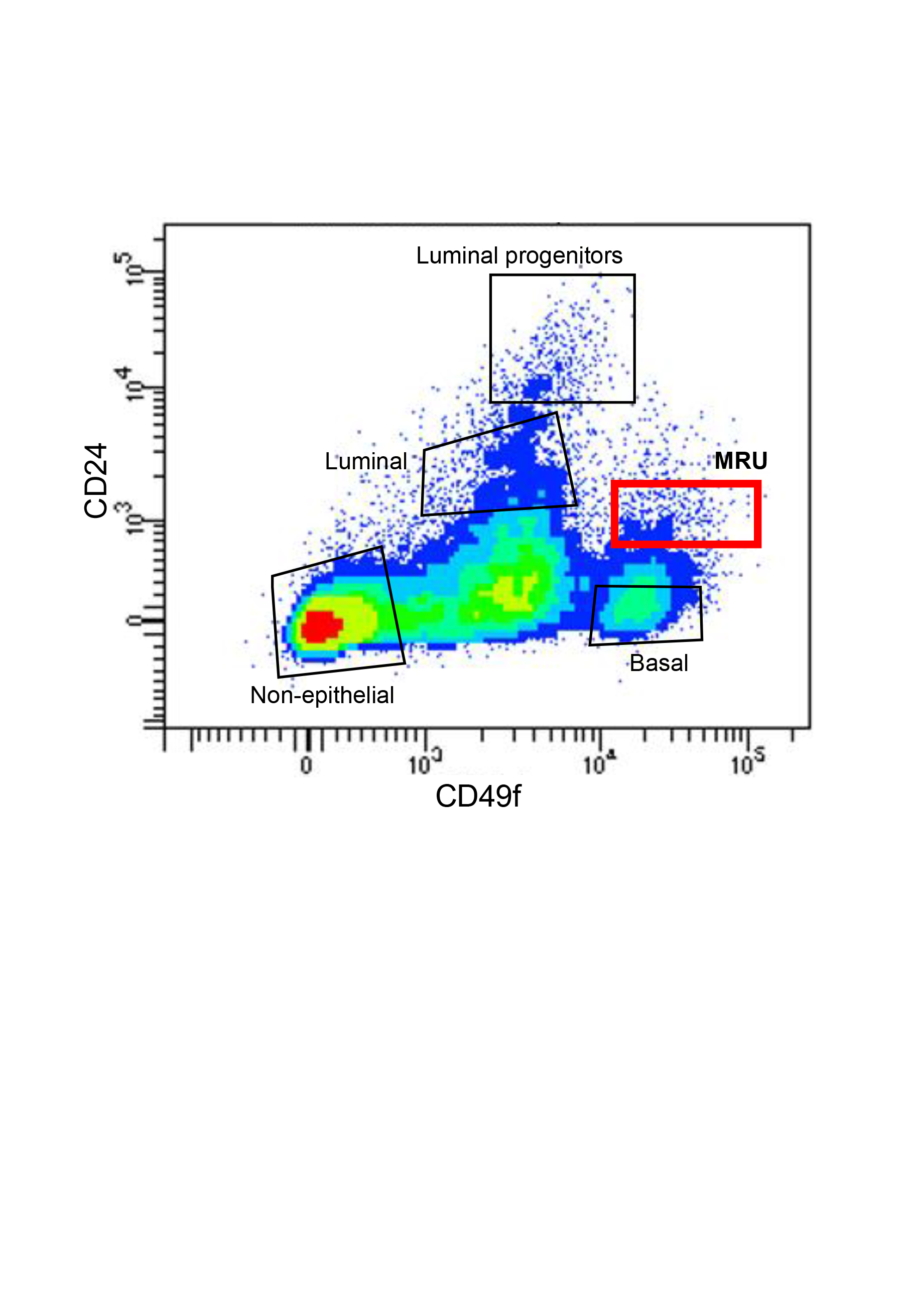

Supplement: S2 Fig — Stem cells (MRU) were sorted from suspensions of single cells of individual vehicle- and rapamycin-treated glands. (TIF) [file pone.0269505.s006.tif]

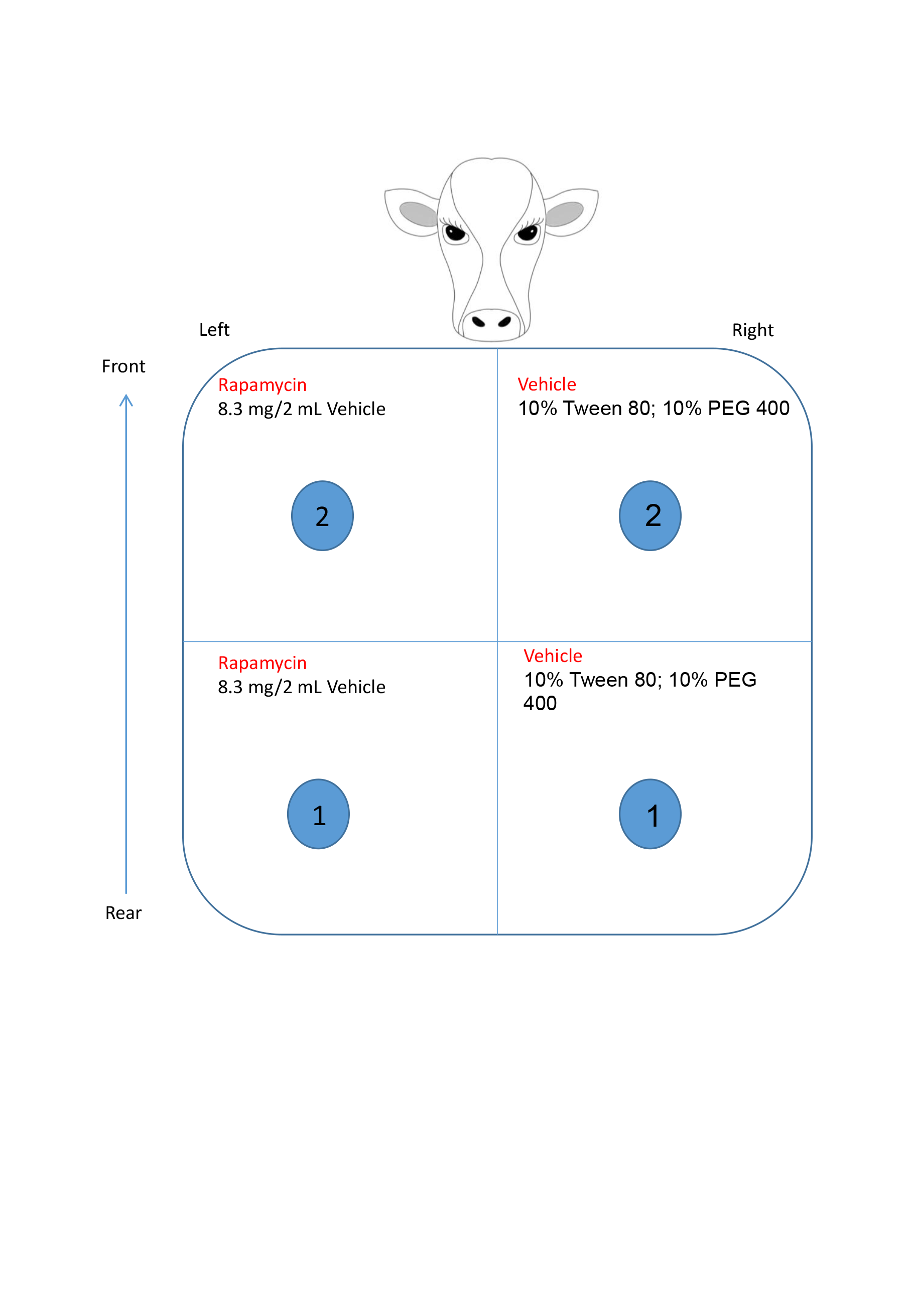

Supplement: S3 Fig — (TIF) [file pone.0269505.s007.tif]

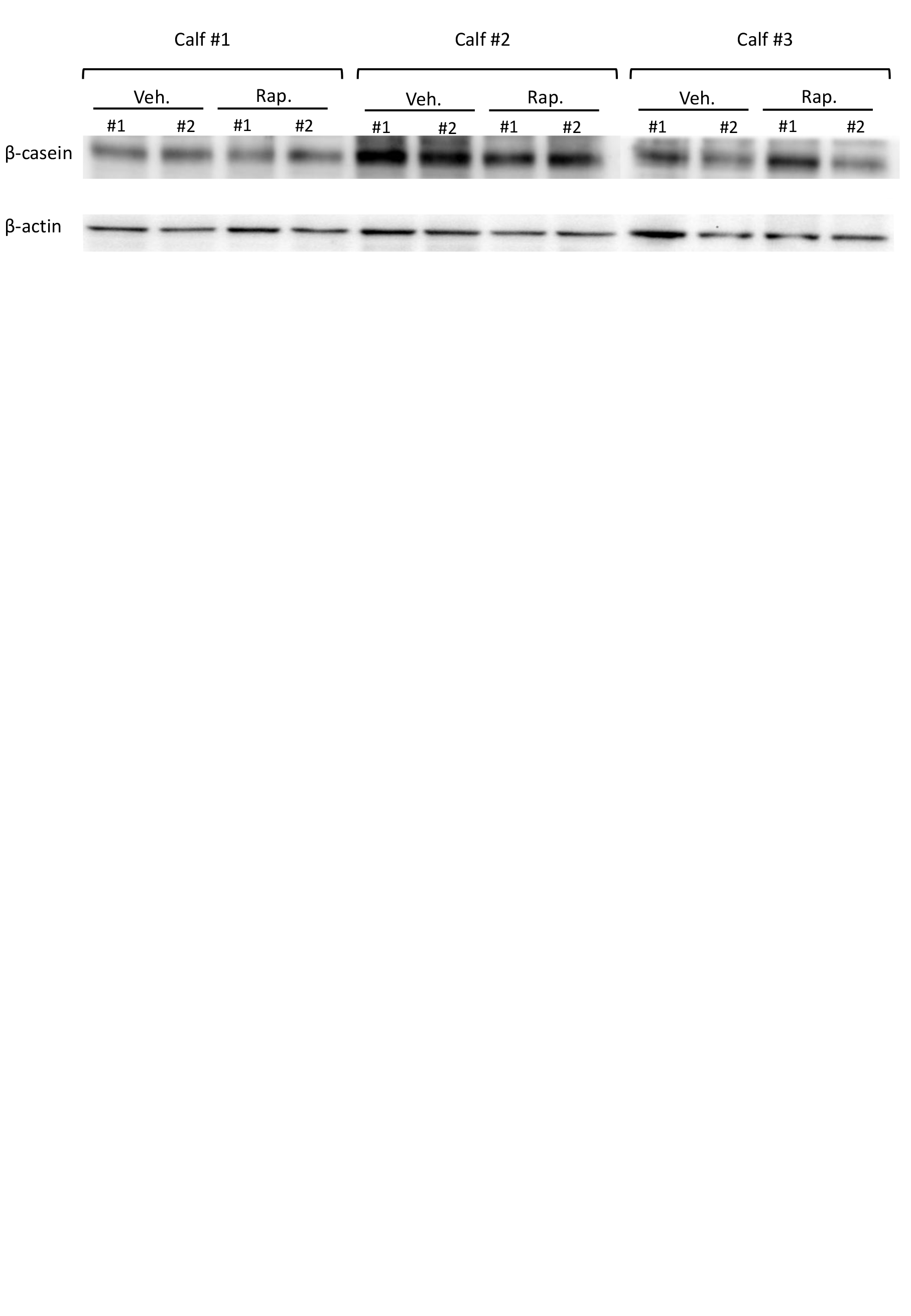

Supplement: S4 Fig — (TIF) [file pone.0269505.s008.tif]

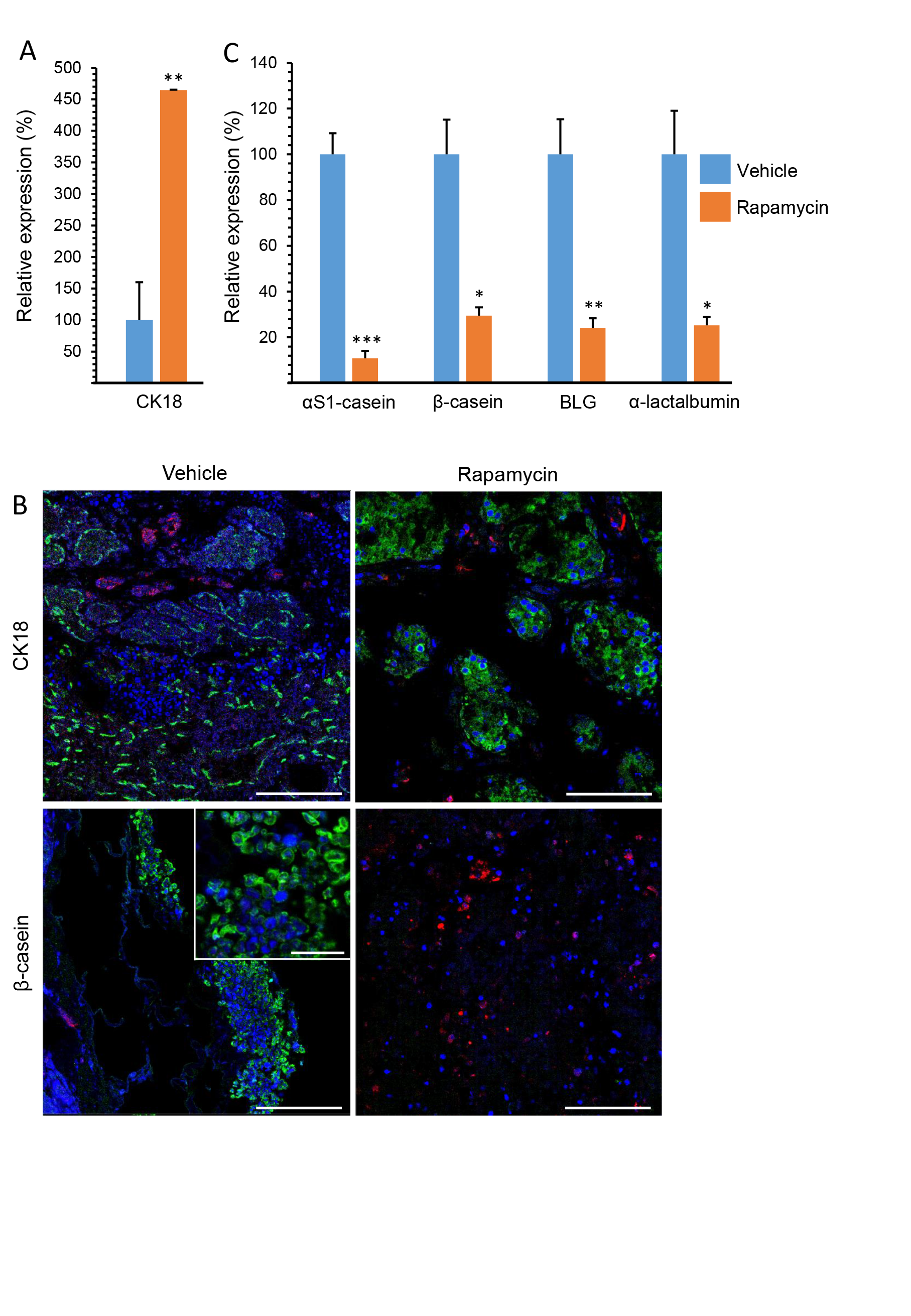

Supplement: S5 Fig — Mammary organoids were cultured for 3 weeks in mammary medium supplemented, or not, with rapamycin. Rapamycin was omitted from the medium for an additional week, then the medium was changed to DMEM/F12 containing insulin, hydrocortisone and prolactin. A. Latent induction of CK18 relative expression by rapamycin administration. B. Immunofluorescence analysis demonstrating induced CK18 expression and decreased β-casein expression in rapamycin-treated organoids. Green: CK18 and β-casein. Red: αSMA. Bar = 20 μm. Inset: higher magnification. C. Negative effect of rapamycin on milk protein gene expression. Bars represent mean ± SEM of 4 replications. *P < 0.05, **P < 0.01, ***P < 0.001. (TIF) [file pone.0269505.s009.tif]
