# Supplementary material for: Intramammary rapamycin administration to calves induces epithelial stem cell self-renewal and latent cell proliferation and milk protein expression
Source: PLoS One. 2022 Jun 22;17(6):e0269505. doi: 10.1371/journal.pone.0269505 (PMC9216576; doi:10.1371/journal.pone.0269505)
Supplement: S1 Table — (DOCX) [file pone.0269505.s002.docx]

| Antigen | Primary  antibody | Manufacturer | Dilution | Application | Secondary  antibody | Manufacturer | Dilution |
| --- | --- | --- | --- | --- | --- | --- | --- |
| αSMA | Mouse  monoclonal, sc-32251 | Santa Cruz  Biotechnology  Santa Cruz, CA | 1:75 | IF | Cy3-conjugated  donkey anti-mouse  IgG | Jackson  ImmunoResearch | 1:100 |
| β-casein | Rabbit polyclonal | Barash lab. | 1:100  1:500 | IHC   WB | Histofine,  H1906-1  Donkey anti-rabbit NA934V | Jackson  ImmunoResearch  GE Healthcare | 1:400   1:5000 |
| pS6 | Rabbit polyclonal | Cell signaling Inc. Danvers, MA | 1:100  1:750 | IHC  WB | Histofine,  H1906-1  Donkey anti-rabbit NA934V | Nichirei Biosciences Inc.  GE Healthcare | 1:5000 |
| S6 | Rabbit monoclonal | Cell signaling Inc. Danvers, MA | 1:1000 | WB | donkey anti-rabbit NA934V | GE Healthcare | 1:5000 |
| CK18 | Rabbit polyclonal, GTX112978 | GeneTex Hsinchu City, Taiwan, R.O.C | 1:100 | IF  IHC | Alexa Fluor  488-conjugated  goat anti-rabbit  IgG   Histofine,  H1906-1 | Jackson  ImmunoResearch  Nichirei Biosciences Inc.. | 1:400 |
| PCNA | Mouse  monoclonal, clone PC10 | BioLegend  San Diego, CA | 1:200 | IHC | Histofine,  H1906-1 | Nichirei Biosciences Inc. |  |
| β-actin | Rabbit polyclonal | Cell signaling Inc. Danvers, MA | 1:1000 | WB | Donkey anti-rabbit NA934V | GE Healthcare | 1:5000 |

S1 Table. List of antibodies used in this study
