# Supplementary material for: Intramammary rapamycin administration to calves induces epithelial stem cell self-renewal and latent cell proliferation and milk protein expression
Source: PLoS One. 2022 Jun 22;17(6):e0269505. doi: 10.1371/journal.pone.0269505 (PMC9216576; doi:10.1371/journal.pone.0269505)
Supplement: S2 Table — (DOCX) [file pone.0269505.s003.docx]

| Gene | Gene ID | Forward primer (5’→3’) | Reverse primer (5’→3’) | Product  size (bp) |
| --- | --- | --- | --- | --- |
| α-Casein S1 | 282208 | AAGAGGGAATCCATGCCCAA | AGGCCAGTTCCTGATTCACTC | 60 |
| DELTA 1 (DLL1) | 788775 | AGGGCCAGTACTGCACAGA | AAAATCCGTGCTGCTCATC | 60 |
| ERα (ESR1) | 407238 | CGCAAGTGCTATGAAGTGG | CGCTTGTGCTTCAACATTC | 80 |
| STAT5A | 282375 | GCAGCCATCTCGAGGACTA | GAACCACTGCCAGAAGGTG | 98 |
| α-lactalbumin | 281894 | CTCCAGGGGTGCATGAATGG | TAAAAGCGCCATCAGGGACAT | 67 |
| BLG | 100848610 | GGGAAAACCACGAGTGTGTG | TTTCCCCGTGATAGTTGACCG | 87 |
| EIF4E | 281751 | CAGGAGGTTGCTAACCCAGA | TGCTTGCCAAGTTTTGCTT | 99 |
| Ki67 | 513220 | GTCAGCAGCTTCGGTGATT | GAGACCCGCCTCCTCTTT | 65 |
| Notch1 | 767866 | AACGAGTTCGTGTGCGAGT | GTTCTTGCAGGGTGTGCTT | 90 |
| Jagged 1 | 783681 | TCCCACTGGTTTCTCTGGA | CTGGCTCGGTTGTAGCACT | 96 |
| LGR4 | 505423 | GGGCTTAAAAGCACCTGTCGAA | AGGTCTCAGTGATGTGGCAGG | 100 |
| LGR5 | 520189 | AGTTGTTCAGCCTCCGATCT | TGGAAAATGCATTAGGGTCA | 84 |
| S16 | 506297 | ATCTCGTCTCTGCGCCTTGA | GCATTCTTCTGCCGGTGTCG | 85 |
| LGR6 | 100336662 | GTGTCCTGGAGCTGTCTCAC | CTCCTCCAGCTTCTGACACC | 71 |
| β-casein | 281099 | TTCCATTGCCTGGACTACTTGT | CAACAGCCAAAAGGCAGGTA | 90 |

**S2 Table.** List of primers used in this study
