# Supplementary material for: Intramammary rapamycin administration to calves induces epithelial stem cell self-renewal and latent cell proliferation and milk protein expression
Source: PLoS One. 2022 Jun 22;17(6):e0269505. doi: 10.1371/journal.pone.0269505 (PMC9216576; doi:10.1371/journal.pone.0269505)
Supplement: S3 Table — The data indicate no significant effect for individual heifer or gland. (DOCX) [file pone.0269505.s004.docx]

| Calf | Gland^1^ | Level total cell | Cell number | Level diameter | Diameter |
| --- | --- | --- | --- | --- | --- |
| #1 | Vehicle #1 | A | 79.76190 | A | 111.17268 |
|  | Vehicle #2 | A | 97.78947 | A | 110.06925 |
|  | Rapamycin #1 | A | 77.07692 | A | 129.88807 |
|  | Rapamycin #2 | A | 71.95833 | A | 99.97315 |
| #2 | Vehicle #1 | A | 94.53571 | A | 109.03316 |
|  | Vehicle #2 | A | 84.85000 | A | 126.03119 |
|  | Rapamycin #1 | A | 102.44444 | A | 123.61209 |
|  | Rapamycin #2 | A | 89.66667 | A | 106.04873 |
| #3 | Vehicle #1 | A | 65.70588 | A | 105.37013 |
|  | Vehicle #2 | A | 77.47619 | A | 106.85253 |
|  | Rapamycin #1 | A | 74.39130 | A | 103.89836 |
|  | Rapamycin #2 | A | 87.90476 | A | 113.73072 |

**S3** **Table.** Tukey–Kramer (means comparison) analysis of average cell number/duct and average ductal diameter in mammary glands treated with vehicle or rapamycin for 3 weeks followed by estrogen and progesterone. The data indicate no significant effect for individual heifer or gland.

^1^ For identification of the gland’s position, see Figure S3.
