# Supplementary material for: Intramammary rapamycin administration to calves induces epithelial stem cell self-renewal and latent cell proliferation and milk protein expression
Source: PLoS One. 2022 Jun 22;17(6):e0269505. doi: 10.1371/journal.pone.0269505 (PMC9216576; doi:10.1371/journal.pone.0269505)
Supplement: Attachment — Submitted filename: PONE-D-22-05135_reviewers comment.pdf [file pone.0269505.s010.pdf]

# Intramammary rapamycin administration to calves induces epithelial stem cell self-renewal and latent cell proliferation and milk protein expression

Anna Kosenko, Shamay Jacobi, Tomer-Meir Salame, Maya Ross and Itamar Barash*

Anna et al, in this study, have perused the effects of inhibition of mTOR activity in inducing stem cell population in bovine mammary tissue using calves as a model animal. The investigators are working to enhance cell proliferation in mammary and intestinal stem cells, including intramammary administration of xanthosine, inosine, calorie restriction, and decreasing mTOR activity. The objective of the present study is to suppress mTOR activity in the calf mammary gland by rapamycin signals and see its effects on mammary stem cell self-renewal. This study extended the investigator's mice-model analysis of bovine mammary implants and showed improved mammary development upon rapamycin administration. The data supported investigators of the earlier research and showed latent rapamycin-induced luminal and basal/myoepithelial cell proliferation to be the cell-type and location-specific. The authors concluded that the positive latent effects of the inhibitor of mTOR, rapamycin, may be beneficial in cellular manipulation of bovine mammary epithelial cells for improving milk protein expression in dairy animals. This is an exciting study and worthy of being considered for publication upon making suggestive changes.

**Major issues**

1. Line 72-73: A study suggesting the role of xanthosine in increasing stem cell population is not limited to bovine only but has similar effects in the goat mammary gland (Choudhary et al., 2018; https://pubmed.ncbi.nlm.nih.gov/29804277/). Reference should be mentioned here.
2. Line 183: Please elaborate on how protein concentration in the sample was determined to have an equal amount of protein per sample loaded for SDS-PAGE.
3. Line 416-17: The presence of differentiated mammary epithelial cells in 3- months old calf is engaging. This information should be discussed a bit more here. This result is consistent with an earlier study where expression of milk protein genes was evident in the RNAseq study in the prepubertal buffalo mammary gland (Choudhary et al. 2019; https://pubmed.ncbi.nlm.nih.gov/30467802/). Please discuss this point here.
4. Figure 2E: Please provide a representative picture of S6P staining of mammary tissue under the "Rapamycin" group. It is harder to visualize any cellular architecture of the tissue.
5. Figure 5B: Please provide a representative picture of the under Rapamycin group showing a comparatively high number of PCNA-positive epithelial cells. It is challenging to visualize the increased cell proliferation rate in the rapamycin group.

**Minor issues**

- Line 638-639 and line 648-649: Please check if the information (duct no, cell no) is not the same in the legend of fig 4 and fig 5.
-
